# The Development of Light-Curable Calcium-Silicate-Containing Composites Used in Odontogenic Regeneration

**DOI:** 10.3390/polym13183107

**Published:** 2021-09-15

**Authors:** Yi-Ting Lin, Ming-You Shie, Yen-Hong Lin, Chia-Che Ho, Chia-Tze Kao, Tsui-Hsien Huang

**Affiliations:** 1 School of Dentistry, Chung Shan Medical University, Taichung 40201, Taiwan; ytdoctor@gmail.com (Y.-T.L.); ctk@csmu.edu.tw (C.-T.K.); 2 School of Dentistry, China Medical University, Taichung 406040, Taiwan; eric@mail.cmu.edu.tw; 3 x-Dimension Center for Medical Research and Translation, China Medical University Hospital, Taichung 404332, Taiwan; 4 Department of Bioinformatics and Medical Engineering, Asia University, Taichung 41354, Taiwan; sethho@asia.edu.tw; 5 The Ph.D. Program for Medical Engineering and Rehabilitation Science, China Medical University, Taichung 406040, Taiwan; roger.lin0204@gmail.com; 6 Department of Stomatology, Chung Shan Medical University Hospital, Taichung 40201, Taiwan

**Keywords:** calcium silicate, polyethylene glycol diacrylate, light curing, dental pulp stem cells, odontogenesis

## Abstract

Pulp regeneration is one of the most successful areas in the field of tissue regeneration, despite its current limitations. The biocompatibility of endodontic biomaterials is essential in securing the oral microenvironment and supporting pulp tissue regeneration. Therefore, the objective of this study was to investigate the new light-curable calcium silicate (CS)-containing polyethylene glycol diacrylate (PEGDA) biocomposites’ regulation of human dental pulp stem cells (hDPSCs) in odontogenic-related regeneration. The CS-containing PEGDA (0 to 30 wt%) biocomposites are applied to endodontics materials to promote their mechanical, bioactive, and biological properties. Firstly, X-ray diffraction and Fourier-transform infrared spectroscopy showed that the incorporation of CS increased the number of covalent bonds in the PEGDA. The diameter tension strength of the CS-containing PEGDA composite was significantly higher than that of normal PEGDA, and a different microstructure was detected on the surface. Samples were analyzed for their surface characteristics and Ca/Si ion-release profiles after soaking in simulated body fluid for different periods of time. The CS30 group presented better hDPSC adhesion and proliferation in comparison with CS0. Higher values of odontogenic-related biomarkers were found in hDPSCs on CS30. Altogether, these results prove the potential of light-curable CS-containing PEGDA composites as part of a ‘point-of-care’ strategy for application in odontogenesis-related regeneration.

## 1. Introduction

The main purpose of endodontic surgery is to seal tooth defects with suitable root canal filling materials to prevent bacterial overgrowth and colonization [1]. Therefore, bioceramics have been developed and modified to be the first-choice biomaterial for the filling of tooth defects [2]. The ideal root canal filling material should have excellent biocompatibility, bioactivity, sealing capability, X-ray opacity, and antibacterial properties [3]. Of the various bioceramics, mineral trioxide aggregate (MTA) is one of the most widely used filling materials for root canal treatment [4]. MTA is a calcium-silicate-based material that is biocompatible, non-neurotoxic, and does not cause any severe side effects on other organs [5]. In addition, in vivo and clinical studies have confirmed that MTA has a role to play in activating signaling molecules for downstream cellular activities, thus having osteoinductive effects on dental pulp tissues [6]. Today, MTA is also used for tooth discoloration issues, as patient demand for dental aesthetics is increasing. Even though MTA is a popular material for dental applications, a main disadvantage of MTA is that it has a long curing duration of at least 2–5 h. Therefore, although MTA is often used in patients with massive gum bleeding, its long curing duration greatly decreases its sealing performance for bleeding patients [7].

Traditional ceramics are mainly classified as bio-inert ceramics (e.g., alumina, zirconia), absorbable ceramics (e.g., tricalcium phosphate), or biologically active ceramics (e.g., hydroxyapatite and bioglass) [8]. In order to further enhance the biological characteristics of bioceramics, scientists have attempted to load growth factors or related proteins onto bioceramics in order to guide cellular migration to enhance tissue regeneration [9]. Calcium silicate (CS) is another type of bioceramic commonly used in pulp capping or regeneration [10]. Silicon (Si) ions are among the indispensable elements in the development of human bones. Research has found that Si ions are not only responsible for the development of bones and joints, but are also closely related to immune regulation and connective tissue. In addition, Si ions can also induce the formation of calcium matrices, such as bone tissues [11,12,13]. Shie et al. found that a concentration of 4 mM Si ions can enhance bone regeneration via the ERK signaling pathway, and enhance the secretion of type I collagen (Col I) [14]. In addition, some studies have confirmed that Si ions are able to activate the WNT and SHH pathways of bone marrow stromal cells and, thus, promote cell proliferation, differentiation, and expression of osteogenic-related proteins [15]. In our previous studies, we developed CS scaffolds and evaluated them for their potential in bone tissue engineering [16,17]. It was reported that Si ions released from the scaffolds were able to promote odontogenesis of human dental pulp cells and cementogenesis of human periodontal ligament cells [16,18]; in addition, the release of calcium ions from the CS scaffolds facilitated cell attachment and proliferation, enhanced the secretion of growth factors, and promoted bone regeneration. Calcium ions are known to combine with phosphate radicals to form hydroxyapatite (HA) on the surfaces of CS scaffolds, and the breakdown of CS byproducts has also been reported to increase the pH of the microenvironment, thus giving it an additional antibacterial tool. It is important to note that the angiogenesis of human umbilical vein endothelial cells is beneficial to the regeneration of hard tissues [19].

At present, the most common light-curable MTA material is mainly composed of MTA ceramic powder mixed with bisphenol A-glycidyl methacrylate (Bis-GMA) [20]. Bisphenol A is derived from Bis-GMA, which is routinely used for the repair of decayed, fractured, and poorly formed teeth [21]. Bisphenol-A can greatly enhance and improve the existing shortcomings of MTA, such as its low strength, lack of resistance, difficulty to handle, and insufficient gloss and polishing ability [22]. However, many studies still point out that bisphenol A is a harmful estrogen endocrine-disrupting substance. In order to solve these issues, trimethylene glycol- or methacrylate-containing Bis-GMA-free biopolymers have been studied. However, the use of endodontic clinical therapy, whether or not it has sufficient physical/chemical properties, is still criticized by users.

In this study, a new light-curable endodontic material incorporating CS powder with polyethylene glycol diacrylate (PEGDA) was examined. This study investigated the chemical and physical properties of different CS ratios of the light-curable endodontic material. Moreover, the cell behaviors—such as the adherence, proliferation, differentiation, and odontogenic-related markers—of human dental pulp stem cells (hDPSCs) cultured on the new light-curable CS material were examined.

## 2. Materials and Methods

### 2.1. Preparation of the Light-Curable CS Specimens

Calcium silicate (CS) powder was prepared according to methods published previously [23]. In brief, reagent-grade calcium oxide (CaO, Sigma-Aldrich, St. Louis, MO, USA), silicon dioxide (SiO_2_, Sigma-Aldrich, St. Louis, MO, USA), and aluminum oxide (Al_2_O_3_, Sigma-Aldrich, St. Louis, MO, USA) powders were weighed and mixed in a ball mill according to specific proportions, after which the mixture was dried in an oven and heated in a 1400 °C high-temperature sintering furnace for 2 h, where the temperature was raised in increments of 10 °C per minute for the purpose of sintering, after which the temperature was then naturally cooled to room temperature.

To make the light-curable composite, CS powder and PEGDA (MW = 700, Sigma-Aldrich, St. Louis, MO, USA) were mixed according to different weight percentages (CS ratio: 0/10/20/30 wt%) with 0.25 wt% lithium phenyl-2, 4, 6-trimethylbenzoylphosphinate (LAP, Sigma-Aldrich, St. Louis, MO, USA) at room temperature in the dark. The specimens were prepared using a light-curing 3D printer (MiiCraft+, MiiCraft™, Hsinchu, Taiwan) according to an STL file prepared using drawing software. The scaffolds were designed to have a diameter of 8 mm and a height of 2 mm for the following tests (except for the diameter tension strength assay), and the curing parameters were set at 90 s per layer (100 µm) using 405 nm UV light.

### 2.2. Physicochemical Properties

X-ray diffraction (XRD, Bruker D8 SSS, Karlsruhe, Germany) was used to analyze the phase structure of the prepared materials. The diffractometer was set at 30 kV and 20° to 60° (2θ) at a rate of 1°/min. In addition, a Fourier-transform infrared spectrometer (FTIR, Vertex 80v, Bruker, Karlsruhe, Germany) was used to analyze the various functional groups in the prepared materials. An EZ-Test instrument (Shimadzu, Kyoto, Japan) was used to evaluate the mechanical properties of the samples by determining their diameter tension strength (DTS). Firstly, the samples were printed into cylindrical shapes with a diameter of 6 mm and a height of 2 mm. A compressive speed of 1 mm/s was applied from above until the specimens were crushed. Six independent scaffolds were prepared for this test, and the test was repeated thrice, with the average recorded. The data were recorded in distance (mm) and load (N), and the corresponding stress–strain graph is presented in our results. To observe the surface morphology, the specimens were first dried, then dehydrated in ethanol and coated with platinum prior to observation. A scanning electron microscope (SEM, JEOL JSM-7800F, Tokyo, Japan) at an acceleration voltage of 20 kV was used to observe the surface topography of the specimens.

### 2.3. Swelling Analysis

To determine the degree of the swelling behavior in the light-curable CS, different concentrations of CS-containing specimens were fabricated with a determined diameter of 12 mm and a thickness of 1 mm, and cured under UV lighting, with uncured material being removed with phosphate-buffered saline (PBS, Invitrogen, Carlsbad, CA, USA). Afterwards, the light-curable CS specimens were dried at 60 °C and weighed again to obtain the dry weight (Wd) of each specimen. Then, the specimens were immersed in PBS for different time intervals and removed from the PBS, dried using filter paper, and weighed to obtain the wet weight (Ws) of the samples, after which the swelling ratio was calculated using the following formula:

(1) $$\mathrm{Swelling}\;\mathrm{ratio}\left(\%\right)=\frac{\left(\mathrm{Ws}–\mathrm{Wd}\right)}{\mathrm{Wd}}\times 100\%$$

### 2.4. In Vitro Immersion Test

The samples were immersed in simulated body fluid (SBF) for this test. The contents of the SBF were as follows: 7.9949 g of NaCl, 0.2235 g of KCl, 0.147 g of K_2_HPO_4_, 0.3528 g of NaHCO_3_, 0.071 g of Na_2_SO_4_, 0.2775 g of CaCl_2_, and 0.305 g of MgCl_2_·6H_2_O. The contents were dissolved in 1000 mL of distilled water in a specific order, and tris buffer and HCl were used to adjust the pH to 7.4. Each sample was immersed in a centrifuge tube containing 50 mL of SBF at 37 °C for various durations. Degradation was presented as the change in weight percentage (∆%), as measured with a balance. The test was repeated thrice, with the average recorded according to ISO 6876. The samples were dried, immersed in SBF, and weighed to obtain an initial weight. After various periods of immersion, the samples were removed, dried, and weighed to obtain the second weight after immersion. The following formula was used to calculate for the degradation rate:

Degree of degradation (%) = [(weight at time − initial weight)/initial weight] × 100(2)

In addition, inductively coupled plasma atomic emission spectroscopy (ICP-AES, PerkinElmer OPT 1MA 3000DV, Shelton, CT, USA) was used to measure the Ca and Si ions released after periods of immersion.

### 2.5. Cell Viability and Morphology

For the cell viability and morphology assay, the cell seeding was performed in the as-prepared samples after 75% EtOH treatment for 30 min and washing 3 times with PBS. The hDPSCs used in this study were purchased from Lonza (PT-5025, Lonza, Basel, Switzerland) and cultured with a commercially available human dental pulp stem cell bullet kit (PT-3005, Lonza, Basel, Switzerland) to passage 4–7 in a 37 °C humidified atmosphere with 5% CO_2_. The hDPSCs were trypsinized using TrypLE™ (Invitrogen, Grand Island, NY, USA), collected using a hemocytometer, and seeded on different light-curable CS specimens at a density of 5 × 10^4^ cells/mL in a 48-well plate with 1 mL of medium per well. The cells were seeded in the cell suspension, along with medium cultured on the surface of the specimens. After culturing for different time intervals, PrestoBlue assay (Invitrogen, Grand Island, NY, USA) was used to evaluate the proliferation of hDPSCs cultured on these samples. In brief, after 1, 3, 5, and 7 days of culture, the medium was mixed with PrestoBlue reagent at a ratio of 9:1, and then added to the culture well for 50 min in a 37 °C incubator. Next, 100 μL of the solution was transferred to new 96-well plate. For this study, the hDPSCs cultured on plates were used as a control group (Ctl). The optical density of the solution was assessed at a wavelength of 570 nm (reference wavelength of 600 nm) using a spectrophotometer (Infinite Pro M200, Tecan, Männedorf, Switzerland).

To observe the cell morphology of the light-curable CS specimens, the specimens were washed several times with cold PBS and fixed in 1.5% glutaraldehyde (Sigma-Aldrich, St. Louis, MO, USA) for 4 h after 1 day of seeding. The samples were then dehydrated using a graded ethanol series for 15 min at each concentration, and dried with liquid CO_2_ using a critical point dryer device (LADD 28000; LADD, Williston, VT, USA). The dried specimens were mounted on holders, coated with gold, and viewed by SEM. In addition, a cytoskeleton observation was conducted using fluorescent staining. The cells cultured on specimens for 1 and 3 days were washed with cold PBS several times and fixed with 4% paraformaldehyde (Sigma-Aldrich, St. Louis, MO, USA) for 20 min at room temperature. The samples were permeabilized with 0.1% Triton X-100 (Sigma-Aldrich, St. Louis, MO, USA) in PBS for 15 min. The fluorescent staining was performed by incubating the specimens with phalloidin conjugated to Alexa Fluor 488 (1:500, Invitrogen, Grand Island, NY, USA) for 2 h in the dark. Then, the specimens were gently rinsed with cold PBS solution to remove excess solution, and DAPI fluorescent dye (Invitrogen, Grand Island, NY, USA) was used and left to react for 20 min in the dark. The specimens were then removed, washed thrice with PBS, and the confocal microscope (Leica TCS SP8, Wetzlar, Germany) was used to observe the cell morphology of the hDPSCs.

### 2.6. Odontogenesis Differentiation Assay

In order to evaluate the levels of odontogenesis differentiation, all specimens loaded with hDPSCs cells were cultured in a differentiation-promoting culture medium (StemPro™ osteogenesis differentiation kit, Invitrogen, Grand Island, NY, USA) for 3, 7, and 14 days, after which the cell-adhered specimens were immersed in NP40 cell lysis solution (Sigma-Aldrich, St. Louis, MO, USA) and centrifuged at 6000 rpm for 15 min. Then, pNPP (Sigma-Aldrich, St. Louis, MO, USA) was used to evaluate the alkaline phosphatase (ALP) activity. Each sample was mixed with pNPP and 1 M diethanolamine buffer for 30 min before the addition of 5 M NaOH to terminate the reaction. The absorbance of each sample was analyzed under 405 nm wavelength light with a spectrophotometer. All data were standardized with references according to the protein quantitative detection reagents (BCA, Thermo Fisher Scientific, Waltham, MA, USA). In addition, the production of dentin sialophosphoprotein (DSPP, MBS2022855, MyBioSource, San Diego, CA, USA) and dentin matrix protein-1 (DMP-1, MBS167298, MyBioSource, San Diego, CA, USA) secretion from the hDPSCs was determined using ELISA kits, following the manufacturer’s instructions. The protein concentrations were measured based on correlations with a standard curve. All experiments were performed in triplicate.

### 2.7. Data Analysis

A one-way analysis of variance and Scheffe’s multiple comparisons test were used in this study to evaluate differences between each group and scaffold. A value of <0.05 was considered to be statistically significant.

## 3. Results and Discussion

### 3.1. Synthesis and Characterization of the Light-Curable CS Composite

**Figure 1:**
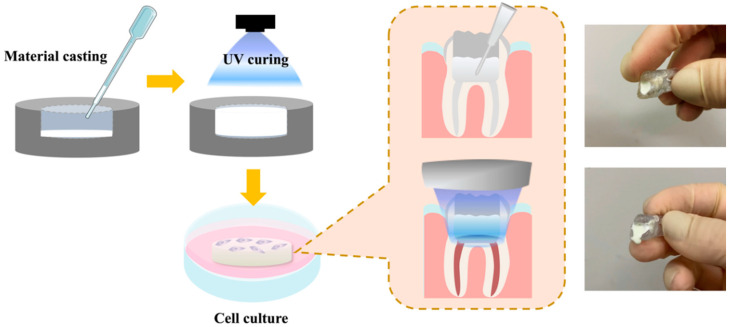
Schematic diagram of fabrication and light-curing of CS-containing PEGDA composites for endodontic applications.

A schematic diagram depicting the fabrication and cell culture of the specimens is shown in Figure 1. Different concentrations of light-curable CS were prepared according to the methods described in the section above. The fabricated light-curable CS composites were then loaded with hDPSCs, cast in pre-fabricated molds, and cured using UV light. This concept arose from the idea that we could inject the light-curable CS into tooth defects of patients, and further cure it using UV light to fabricate personalized scaffolds for unique individuals. CS itself is a synthetic material, thus possessing good mechanical properties, but poor flexibility. By adding a light-curable component to CS, we can implement liquidity and viscosity to it, thus making it injectable, able to take up specific shapes and sizes to act as a tooth filling material. In addition, an image of the light-cured scaffold is shown in Figure 1.

**Figure 2:**
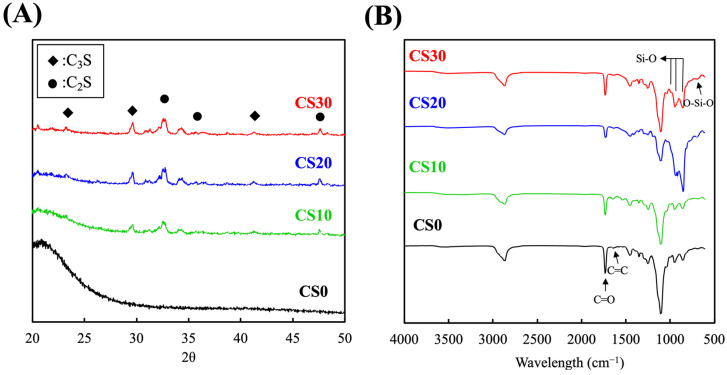
(**A**) X-ray diffractometry and (**B**) Fourier-transform infrared spectroscopy results for the various light-curable CS-containing PEGDA composites.

The XRD patterns of photo-cured PEGDA containing different amounts of CS are revealed in Figure 2A. A strong and broad peak in the 2θ range between 20° and 25° can be seen in the XRD pattern of CS0, indicating the amorphous nature of PEGDA specimens. In contrast, newly formed characteristic peaks located at 29.6° and 32.6°/34.2°, which could be attributed to the presence of tricalcium silicate (C3S) and dicalcium silicate (C2S), respectively, emerged when CS particles were introduced to the PEGDA solution [24]. As expected, the intensities of the peaks corresponding to the calcium silicate were increased, while those corresponding to the PEGDA were decreased, when the CS content in PEGDA was increased from 10 wt% (CS10) to 20 wt% (CS30) [25]. However, a negligible difference was observed while comparing the patterns of CS20 and CS30, which could be attributed to the agglomeration of CS particles. As shown in Figure 2B, characteristic peaks at 2869, 1732, 1638, and 1104 cm^−1^—corresponding to C–H, C=O, C=C, and C–O stretching vibrations, respectively—can be observed in the FTIR spectrum of CS0 [26]. Regarding to the CS/PEGDA groups, it can be clearly observed that the stretching signals of C=O and C=C gradually decreased with increasing CS content, along with the emergence of characteristic peaks at 950 and 858 cm^−1^, attributed to O–Si–O and Si–OH, respectively, while the CS content was not higher than 20 wt% [27]. However, the intensities related to CS decreased when the CS content was increased from 20 wt% to 30 wt%, which may also result from the diminished dispersity of CS in PEGDA. Despite the fact that CS30 was still photocurable, it was considered to be the critical content, owing to the fact that higher content than CS30 may disrupt the integrity of PEGDA, as well as elevating the opacity of the composite, impeding the quantum yield of the photochemical reaction.

### 3.2. The Mechanical Properties and the Swelling Behavior of the Light-Curable CS Composite

**Figure 3:**
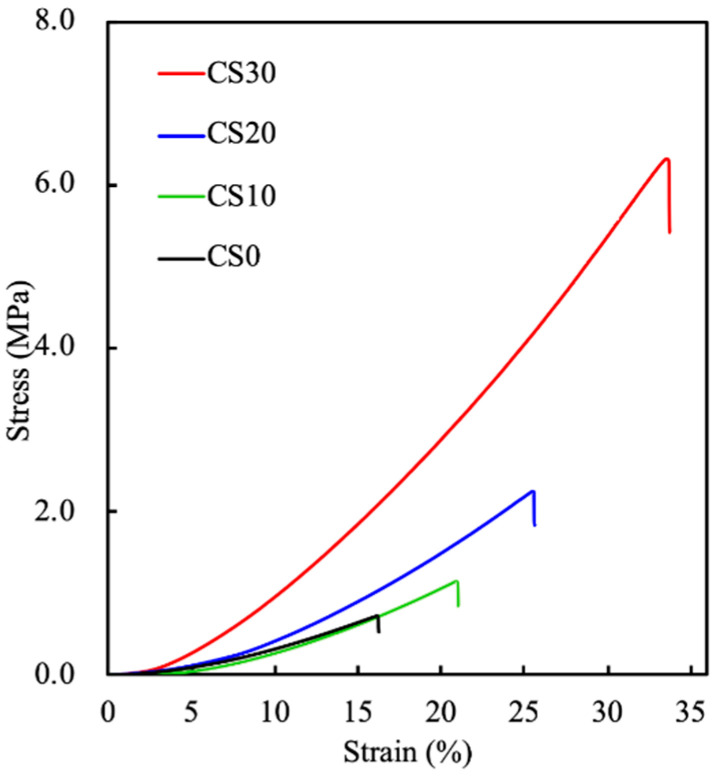
Stress–strain curves of CS0, CS10, CS20, and CS30 composites.

The mechanical strength of dental filling materials is considered to be one of the major attributes that determine the applicable indications in tissue regeneration. [28]. For instance, the pulp capping and coronal restorative materials should possess as much mechanical strength as possible in order to withstand the occlusal load on the restored teeth, whereas the mechanical strength is a minor consideration for root-end filling materials, where minimal loading is exerted [29]. Thus, the mechanical properties of the photocurable PEGDA/CS hydrogels were assessed via a diametral tensile strength test. As seen in the stress–strain curves (Figure 3), the results reveal that the DTS values of CS0, CS10, CS20, and CS30 were 0.72 ± 0.06, 1.13 ± 0.11, 2.22 ± 0.17, and 6.32 ± 0.42 MPa with Young’s moduli of 5.55 ± 0.33, 6.08 ± 0.25, 14.14 ± 0.96, and 23.22 ± 1.42 MPa, respectively, indicating that the presence of CS could be considered as a reinforcing agent to enhance the stiffness of the composite. The mechanical properties including DTS, Young’s modulus, and toughness are summarized in Table 1. Interestingly, a CS-content-dependent enhancement in toughness was observed, of which the toughnesses of CS10, CS20, and CS30 were approximately 2.0, 4.6, and 16.7 times higher than that of CS0. In the present study, the PEGDA and LAP system was selected as the basal component in the photocurable hydrogel, owing to its favorable biocompatibility and rapid photocuring ability [30]. Despite the fact that the mechanical properties of PEGDA can be tailored through tuning the molecular weight and concentration of the prepolymer, type of photoinitiator, and the intensity and exposure time of curing radiation, the fabrication of photocured PEGDA with mechanical strength that matches the clinical requirements of dental and orthopedic applications is challenging. Strategies based on introducing a secondary natural network were evident as an effective route to address this hurdle, and the raised swelling ability may be beneficial to the sealing performance of the filling material [30]; it may also simultaneously enhance the solubility of the composite, due to the degradation of the secondary natural polymer network [31]. Regarding this, CS particles may be considered to be a superior reinforcing agent for developing the photocurable root-filling materials, attributed to their low-solubility nature and superior reinforcing efficiency.

**Figure 4:**
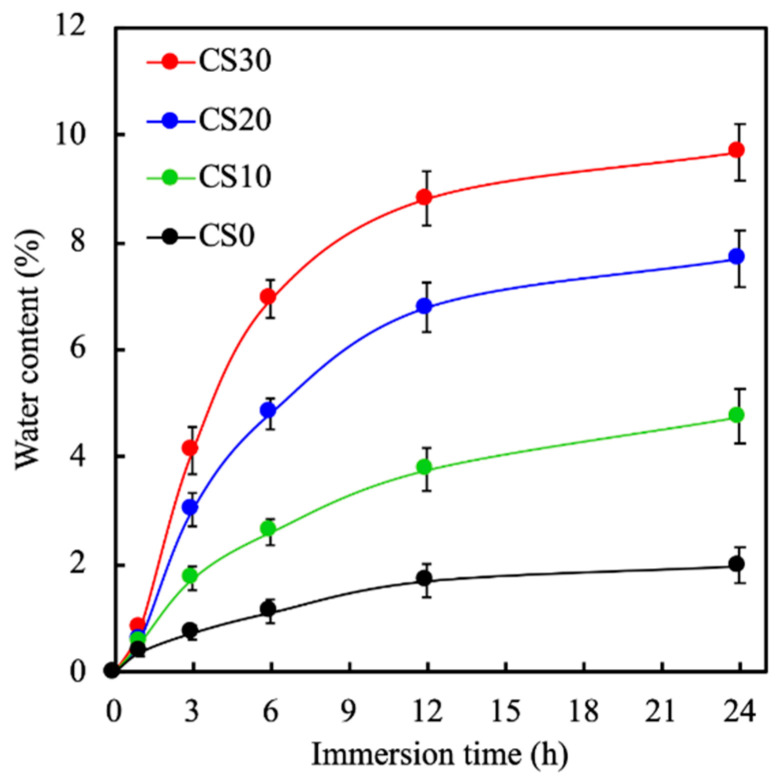
The swelling ratio of light-curable CS-containing PEGDA after immersion in PBS for 24 h. Data presented as mean ± SEM, *n* = 6 for each group.

The swelling rates of the photocured PEGDA containing different amounts of CS were recorded during immersion in PBS for 24 h, and are shown in Figure 4. Equilibrium swelling for all specimens was attained after 12 h of immersion. Swelling capacity was markedly increased for the CS30 in the first 6 h as compared to the rest of the CS composites. All samples were noted to have similar swelling behavior except for their rate of swelling capacity. After 6 h of immersion, CS30 was noted to have approximately 7% water content, as compared to 4.3%, 2.2%, and 0.8% water content for CS20, CS10, and CS0, respectively. In addition, there was no de-swelling noted for any of the scaffolds after 24 h of immersion. The swelling capacity of the dental restorative material depends on the composition and hydrophilicity of the composites. Based on the results above, it can be seen that CS30 had better swelling capability, thus indicating that it also had higher porosity, both of which are important factors for cellular activities.

### 3.3. Effects of Degradation Properties on the Soaking Experiments

**Figure 5:**
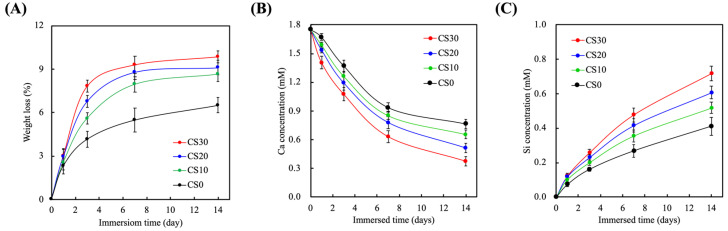
(**A**) The weight loss, (**B**) Ca, and (**C**) Si ions released from the light-curable CS-containing PEGDA composites after soaking in SBF for 1, 3, 7, and 14 days. Data presented as mean ± SEM, *n* = 6 for each group.

The degradation rates of the CS-containing light-curable composites were evaluated by assessing the pre- and post-immersion weights of the specimens, as shown in Figure 5A. As can be seen, the degradation rates of the CS-containing PEGDA varied between the various groups. CS30 showed the highest degradation rate in all groups. All groups displayed rapid degradation during the first 2 and 4 days of immersion, before slowing down to a gradual degradation rate until 14 days of immersion. CS0, -10, -20, and -30 were noted to have a weight loss of 6.5 ± 0.5%, 8.7 ± 0.5%, 9.1 ± 0.6%, and 9.9 ± 0.4% of their total weight, respectively, after 14 days of immersion. In vivo degradation rates are an important factor in determining the ideal biomaterials for tissue regeneration [32]. These biomaterials should ideally match the regeneration rates of tissues in order to provide ample structural support and nutrient transport. Hard tissues typically take 2 weeks to a month for sufficient regeneration; therefore, it was hypothesized that CS30 composites would be able to efficiently support tissue regeneration based on the degradation results above.

The levels of Ca and Si ions released over 14 days of immersion were recorded, and are shown in Figure 5B,C, respectively. As can be seen, CS30 exhibited a gradual decline in Ca release and a gradual increase in Si release over the 14 days of immersion. The levels of Ca and Si ions from the CS30 scaffolds after 14 days of immersion were 0.37 ± 0.05 and 0.72 ± 0.04 mM, respectively, whilst CS0 had 0.76 ± 0.05 and 0.41 ± 0.05 mM of Ca and Si, respectively. It was hypothesized that the decrease in Ca release was due to the pH of the solution used. There are reports stating that Ca release is highest in an acidic environment and lowest in a neutral environment [12]. Ca is mainly found stored in native bones, and has an important role to play in regulating angiogenesis and osteogenesis. It has been reported that Ca concentrations at the lower range of 0.2–0.4 mM facilitate osteoblast proliferation and differentiation, whilst higher concentrations tend to favor extracellular matrix mineralization and remodeling [33]. Ca is reported to regulate bone remodeling via the calcium-sensing receptor (CaSR) and upregulation of insulin-like growth factor II and osteoblastic glutamate. On the other hand, Schwarz et al. first reported on the potential benefits and roles of Si ions in bone tissue regeneration [34]. Si ions are usually absorbed in the form of metasilicate, and are reported to be involved in bone calcification and inhibition of osteoclasts. Recent studies have demonstrated that Si ions are involved in regulating the proliferation and differentiation of stem cells, as well as downstream collagen secretion [35]. Most importantly, it is noted in the present study that the presence of Si ions alone stimulates the osteogenic differentiation of human mesenchymal stem cells in the absence of osteogenic-inducing factors [36]. In addition, the presence of aqueous Si was shown to enhance hydroxyapatite formation on the surfaces of scaffolds, which is known to increase the osteoblast secretion of the extracellular matrix and improve bone–scaffold integration.

**Figure 6:**
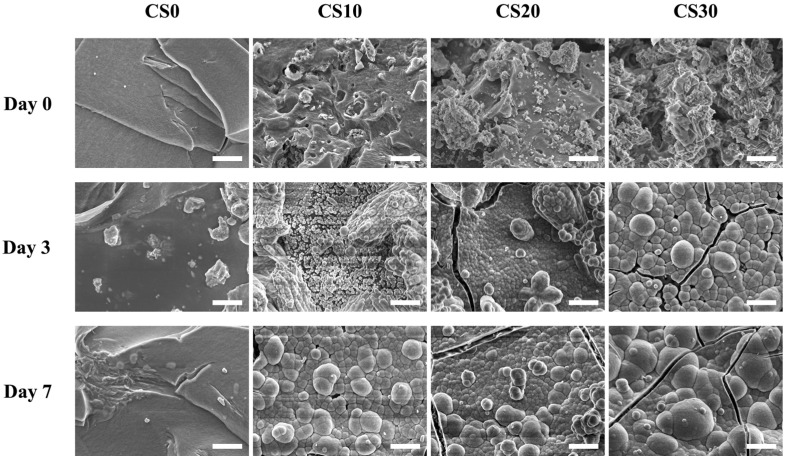
Surface microstructure of the CS0, CS10, CS20, and CS30 composites before and after soaking in SBF for 3 and 7 days. SEM images of the specimens’ surfaces at 10,000× magnification; the scale bar is 2 µm.

CS is known to possess in vitro bioactivity and biocompatibility by precipitation apatite formation with the physiological environment [37]. SEM was used to capture images of the scaffold surfaces after immersion, as shown in Figure 6. On day 0, it could be noted that CS30 had rougher surface contours as compared to CS0 and CS10. It was hypothesized that the rough contours on the surfaces were caused by the addition of CS. Studies have been carried out investigating the effects of surface roughness on cellular behavior [38]. Interestingly, it was reported that the rougher the surfaces, the higher the cellular proliferation and differentiation, due to better cellular adhesion contacts for cells. In addition, clusters of hydroxyapatite agglomerates could be seen on the surface of CS30 after 3 and 7 days of immersion. On the other hand, CS0 had little-to-no hydroxyapatite formation on its surface, whilst the sizes of hydroxyapatite agglomerates were obviously smaller than those on CS30. Hydroxyapatite minerals are known to have similar chemical structures to native bones; therefore, the capacity to induce hydroxyapatite formation on scaffolds is commonly used as an indicator for subsequent bone regeneration. The Ca ions released from CS-containing composites interact with H^+^ ions in the solution, leading to an increase in the SBF’s pH. A series of downstream reactions involving Si, Ca, and P ions resulted in a layer of hydroxyapatite formation on the surfaces of materials [39]. Shie et al. demonstrated that the hydroxyapatite layer enhances the proliferation and differentiation of osteoblast-like MG63 cells, and increases the expression of osteogenic-related genes [40]. Therefore, further tests are required to confirm the odontogenic capabilities of our light-curable CS composites.

### 3.4. In Vitro hDPSCs Culture

**Figure 7:**
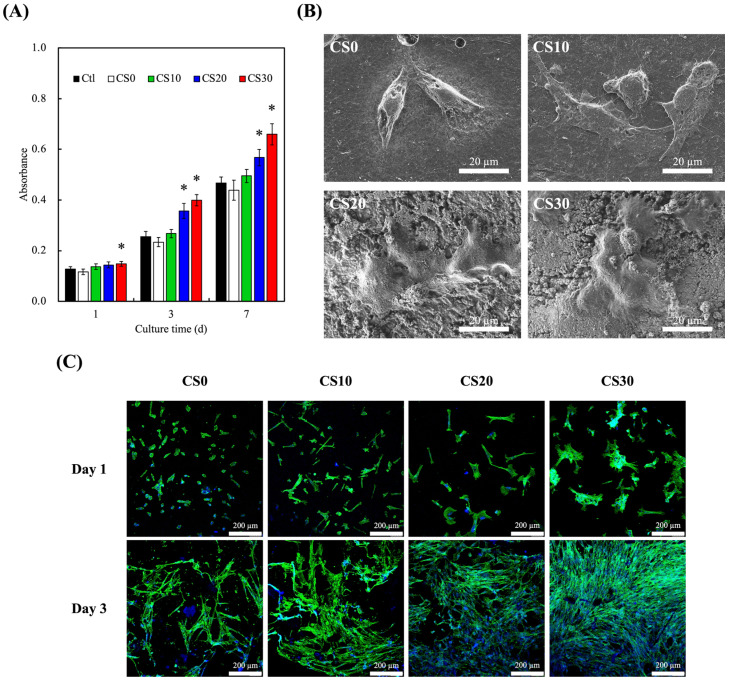
(**A**) Proliferation rate, (**B**) scanning electron microscope images, and (**C**) F-actin (green)/DAPI (blue) staining of hDPSCs cultured on the light-curable CS-containing PEGDA composites for different lengths of time. * indicates a significant difference (*p* < 0.05) from CS0. Data presented as mean ± SEM, *n* = 6 for each group.

The proliferation and cell morphology of hDPSCs cultured with the light-curable CS composites were evaluated, and are shown in Figure 7. After 1 day of culture, CS30 was noted to have significantly higher levels of cellular proliferation as compared to CS0. In addition, CS20 started having significantly higher levels of proliferation from day 3 onwards. However, all groups showed an increase in proliferation in a time-dependent manner. After 7 days of culture, CS30 and CS20 were noted to have 40% and 20% higher proliferation, respectively, as compared to CS0. The SEM micrographs of hDPSCs cultured on different surfaces (Figure 7B) demonstrated that biological adhesion of cells was achieved for all groups within 1 day of culture. However, cells cultured on CS0 and CS10 displayed round shapes despite the existence of filopodia. Contrastingly, specimens with higher CS contents were able to encourage the adhesion of cells, leading to a rather flat and well-spread cell morphology [41]. It is worth noting that the cells were tightly tethered on the apatite adlayer, which was formed as a result of the CS-assisted nucleation of calcium phosphate minerals, as opposed to being directly adhered to the pristine surface as with CS0 and CS10. This implies that the excellent bioactivity and apatite-forming ability of CS are major attributes that were responsible for the prompt achievement of cell adhesion [42]. As can be seen from Figure 7C, hDPSCs cultured on the CS30 composites started to spread after 1 day of culture [43,44]. After 3 days, cell spreading increased considerably for all groups—especially for the CS30 groups. At this stage, it was hypothesized that improved hydrophilicity, mechanical tensile strength, and release of ions played huge roles in enhancing the proliferation of various types of cells [43,45,46]. This revealed that the CS-affected hDPSCs were well-adhered to the surface of the light-curable materials, and were more favorable for cellular proliferation and attachment. Similarly, Mu et al. modified the surface of a 3D-printed Ti scaffold with CS particles, and their results showed that MSCs’ proliferation improved with increased concentrations of CS particles [47]. Both Ca and Si are potent regulators of cellular behaviors, including proliferation and differentiation. Specifically, Zhou et al. reported that the presence of CS bioactive ceramics significantly increased mRNA transcript levels of cyclin B1 and E, which led to a major shift in the cell cycle from the G0/G1 to the S and G2/M phases, thus leading to increased cellular proliferation [48]. These results indicate the potential beneficial effects of the light-curable CS composites used in endodontics engineering, including hDPSCs, bioactive materials, and growth factors involved in odontogenesis [49].

### 3.5. Odontogenic Behaviors

**Figure 8:**
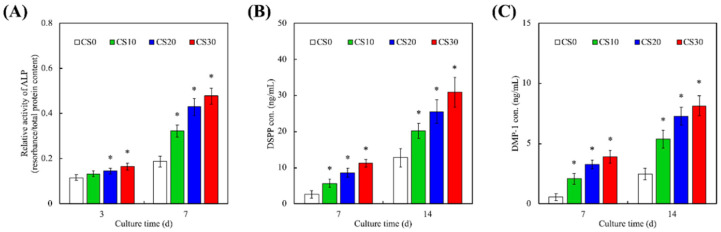
Odontogenesis-related differentiation markers of (**A**) ALP, (**B**) DSPP, and (**C**) DMP-1 expression of hDPSCs cultured on different light-curable CS-containing PEGDA composites for different lengths of time. * indicates a significant difference (*p* < 0.05) from CS0. Data presented as mean ± SEM, *n* = 6 for each group.

The effects of light-curable CS on the expression of ALP, DSPP, and DMP-1 in hDPSCs were investigated, and are shown in Figure 8. In addition, the ECM of teeth consists mainly of collagens and non-collagenous proteins, such as glycoproteins and proteoglycans, of which ALP, DSPP, and DMP-1 make up the bulk of the glycoproteins group [50]. Interestingly, except for day 3 of ALP expression, CS10, CS20, and CS30 exhibited significantly increased expression of ALP, DSPP, and DMP-1 compared to CS0. After 7 days of culture, CS10, CS20, and CS30 were noted to have 50%, 65%, and 85% higher levels of ALP, respectively, as compared to CS0. ALP is considered to be an early marker of initial osteoblast differentiation, and is induced by the presence of Si and Ca ions, as described above. In addition, after 14 days of culture, CS10, CS20, and CS30 were noted to have 40%, 80%, and 110% higher levels of DSPP as compared to CS0. Similar trends were noted for DMP-1, which was mainly involved in dentin remodeling [5]. Dentinogenesis is a process whereby mesenchymal stem cells migrate to the damaged site and differentiate into non-collagenous proteins and collagen-secreting odontoblasts [51]. Amongst the numerous non-collagenous proteins, DSPP is considered to be one of the most critical proteins involved. DSPP is a member of the small integrin-binding ligand N-linked glycoprotein family, whose members share common biochemical characteristics, such as an Arg–Gly–Asp motif. DSPP expression is cell- and tissue-specific, and is seen in high concentrations in odontoblasts and dentine. DSPP is further cleaved into DMP-1, which is involved in downstream intracellular signaling via the mitogen-activated protein kinase and focal adhesion kinase ERK pathways [18]. Taken together, it could be demonstrated that our light-curable CS hydrogels could be the next step in future dentin regeneration applications.

## 4. Conclusions

In this study, a new light-curable calcium silicate powder incorporated with PEGDA was examined. As shown above, the XRD and FTIR demonstrated that CS was successfully incorporated into the PEGDA via covalent bonds. The current results indicate that CS30 can have better mechanical properties than PEGDA (CS0). On the other hand, the stable release mode of Ca and Si ions can also enhance the bioactivity of PEGDA, which can promote the precipitation of apatite on the surface of CS-containing PEGDA in the in vitro immersion experiment. The in vitro cell culture studies indicated that CS30 composites were also beneficial for hDPSCs’ cell behaviors. In particular, CS30 composites exhibited excellent differentiation ability that enhanced the expression of odontogenic-related markers—such as ALP, DSPP, and DMP-1—in hDPSCs. We propose that CS-containing PEGDA composites fabricated via the light-curing technique are extremely advantageous for odontogenesis and pulp regeneration in the challengeable thin-wall dental health. Based on the above results, we are convinced that research on translational medicine for endodontic regeneration therapies involving light-curable CS-based PEGDA composites is also very promising in the near future, as well as for clinical applications.

## Figures and Tables

**Table 1:** Mechanical properties of the light-curable CS composites.

	CS0	CS10	CS20	CS30
Yield strength (MPa)	0.72 ± 0.06	1.13 ± 0.11	2.22 ± 0.17	6.32 ± 0.42
Young’s modulus (MPa)	5.55 ± 0.33	6.08 ± 0.25	14.14 ± 0.96	23.22 ± 1.42
Toughness (J·m^−3^)	4.36	8.56	21.31	84.83

## Data Availability

Data are available in a publicly accessible repository.
